# Nonlinear coherent heat machines

**DOI:** 10.1126/sciadv.adf1070

**Published:** 2023-01-06

**Authors:** Tomáš Opatrný, Šimon Bräuer, Abraham G. Kofman, Avijit Misra, Nilakantha Meher, Ofer Firstenberg, Eilon Poem, Gershon Kurizki

**Affiliations:** ^1^ Department of Optics, Faculty of Science, Palacký University, 17, Listopadu 50, 77146 Olomouc, Czech Republic.; ^2^ Department of Chemical and Biological Physics, Weizmann Institute of Science, Rehovot 7610001, Israel.; ^3^ Physics of Complex Systems, Weizmann Institute of Science, Rehovot 7610001, Israel.

## Abstract

We propose heat machines that are nonlinear, coherent, and closed systems composed of few field (oscillator) modes. Their thermal-state input is transformed by nonlinear Kerr interactions into nonthermal (non-Gaussian) output with controlled quantum fluctuations and the capacity to deliver work in a chosen mode. These machines can provide an output with strongly reduced phase and amplitude uncertainty that may be useful for sensing or communications in the quantum domain. They are experimentally realizable in optomechanical cavities where photonic and phononic modes are coupled by a Josephson qubit or in cold gases where interactions between photons are transformed into dipole-dipole interacting Rydberg atom polaritons. This proposed approach is a step toward the bridging of quantum and classical coherent and thermodynamic descriptions.

## Teaser

Heat engines can work coherently and autonomously without heat baths, using nonlinearly coupled hot and cold light beams.

## INTRODUCTION

A heat engine (HE) may be viewed as a device capable of two functionalities. First, it must concentrate the energy of a heat bath in a selected degree of freedom called the working mode, within limits dictated by the first and second laws of thermodynamics. The enormous number of bath modes characteristic of existing HEs justifies the thermodynamic paradigm, dating back to Carnot, that describes HE as open systems dissipated by thermal baths (*1*–*6*). Second, the concentrated heat must be partly converted into work output, which requires the state of the working mode (*3*–*25*), to store energy in ordered (nonpassive) form, i.e., store ergotropy (*14*, *15*, *26*–*28*). The required nonpassivity/ergotropy is generally achievable either via external control of a piston in HE (*5*–*21*) or via information readout and feedforward by an observer (Maxwell’s demon) in information engines (*29*–*33*). A coherent Maxwell demon engine composed of two qubits in nuclear magnetic resonance setups has been proposed (*34*).

Here, we introduce the concept of a purely coherent, autonomous, closed-system HE, using nonlinear coupling of thermal, continuous-variable, bosonic field modes. This makes these devices fundamentally different from existing HE that is energized by macroscopic baths composed of linearly coupled oscillator modes (*1*–*25*, *33*, *35*–*47*). The proposed nonlinear unitaries are non-Gaussian operations (NGOs) that have been conceived in quantum optical and quantum information schemes (*48*, *49*) but are mostly uncharted terrain in the context of HE, with few exceptions (*18*, *50*).

The envisaged NGO can achieve both HE functionalities discussed above. Thanks to their hitherto unexploited nonlinearity, they can make the output field modes interfere constructively or destructively despite the input phases randomness. These NGOs cause information flow among the modes, resulting in autonomous feedforward of the information, as opposed to externally controlled HE (*3*–*28*, *45*–*47*). In contrast, linear Gaussian operations (LIGOs) are incapable of performing this feat. In quantum optics, LIGOs encompass all energy-conserving linear interference operations caused by beam splitters (BSs) and phase shifters. In contrast, squeezing is neither linear nor energy conserving, but it is a Gaussian operation whose effect on HEs has been studied by us (*15*, *16*, *20*).

The proposed machines are dubbed here as HEs via nonlinear interference (HENLIs). HENLIs lie beyond the present scope of the resource theory of quantum thermodynamics (*51*–*55*) based on LIGO, which relies on energy-preserving joint unitaries performed on quantum systems and their thermal bath ancillae.

## RESULTS

### Minimal HENLI analysis

**Fig. 1.**
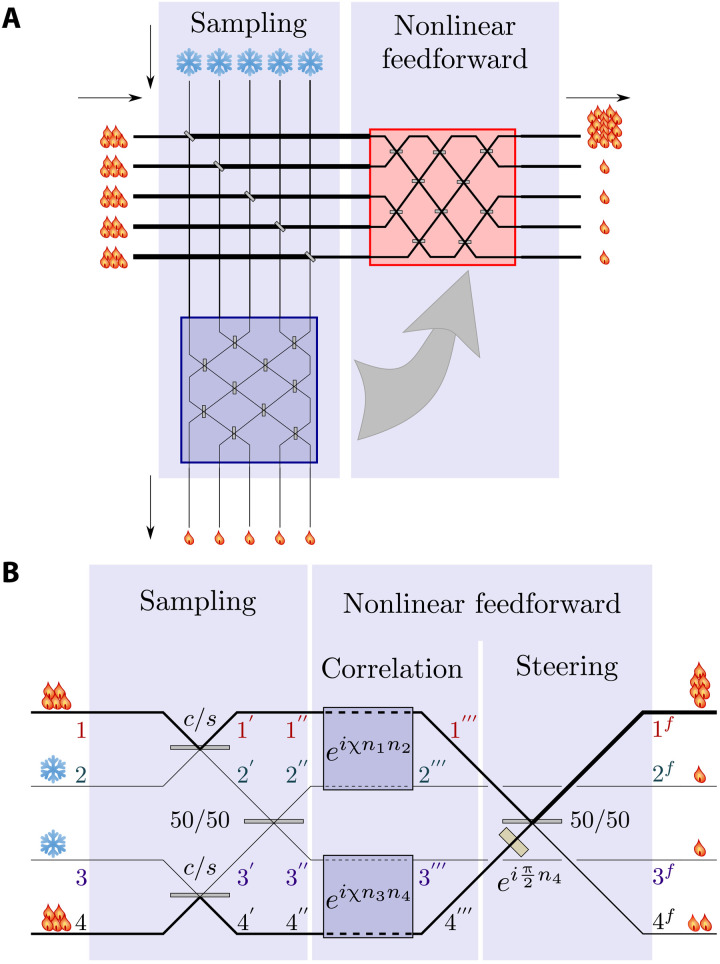
Schematic of the stages of HENLI. (**A**) (i) A fraction of the energy of the hot modes is split off by BSs (in the bottom blue box) to carry information about the remaining hot modes (in the right red box). (ii) Nonlinear interactions correlate these two boxes and autonomously feedforward the information such that (iii) the output fields can be steered to interfere constructively in one preselected mode. (**B**) A four-mode HENLI in which the input modes (1 and 4, hot; 2 and 3, cold) undergo the aforesaid stages (see main text). For optimal parameter choice, the interference is predominantly constructive in mode 1*^f^* and destructive in mode 4*^f^*.

Energy concentration must involve at least two hot modes, and each such mode is sampled (copied) by at least one cold mode. Therefore, the minimal version of HENLI (Fig. 1B) contains two hot and two cold input modes, for simplicity, at the same frequency (nondegenerate mode analysis is laborious and does not reveal essentially new insights). The coherence length of this interferometer should be much longer than its spatial size, so that temporal evolution can be replaced by discrete steps, each described by a unitary evolution operator. We analyze the autonomous feedforward of the a priori unknown amplitudes of the hot modes 1 and 4 and their steering that maximize the energy and work capacity (ergotropy or nonpassivity) (*14*, *15*, *26*–*28*) of mode 1 at the output:

(i) At the sampling stage, the first BS, with low transmissivity *s* = sinθ ≪ 1 (high reflectivity *c* = cosθ ≲ 1), causes small fractions of the hot input modes 1 and 4 to split off and merge, respectively, with the empty modes 2 and 3, so that we have weak copies 2${}^{\prime }$ and 3${}^{\prime }$ of 1 and 4, respectively. For each coherent-state realization, these weak copies have the same phase difference ϕ as the input modes 1 and 4 and mean intensity difference proportional to the mean quanta number difference *n*_. The weak copies then merge on a 50/50 BS whose output modes are 2${}^{\prime \prime }$ and 3${}^{\prime \prime }$.

The correlations generated between *n*_ and ϕ are quantified by the mutual information (MI) (*56*). At the *k*th stage (*57*, *58*), MI is

$$I_{k}=\sum_{n\_}\int_{0}^{2\pi }P_{k}(n\_,\phi )\ln\frac{P_{k}(n\_,\phi )}{p_{k}(n\_){\tilde{p}}_{k}(\phi )}d\phi$$ (1)

where 𝒫*_k_*(*n*_, ϕ) is the joint distribution and *p_k_*(*n*_) and ${\tilde{p}}_{k}(\phi )$ are the marginal distributions. Initially, there is no correlation, i.e., MI vanishes.

**Fig. 2.**
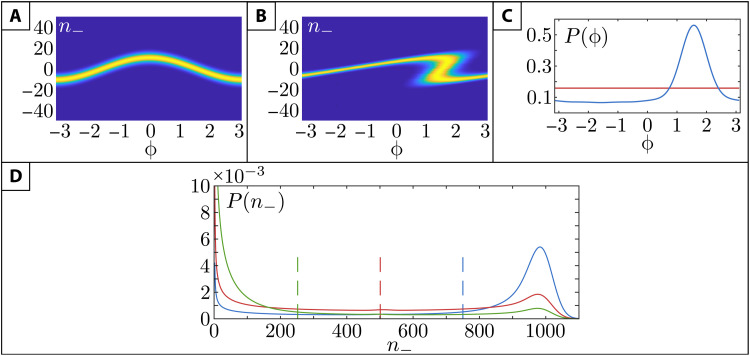
MI and initial and final distribution for HENLI. (**A**) Joint distribution 𝒫(*n*_−_, ϕ) after the sampling stage. Brighter color, higher probability. (**B**) Same distribution after the cross-Kerr stage.(**C**) Phase-difference distribution initial (red) and final (blue). (**D**) Output quanta number distribution in mode 1*^f^* (Eq. 3) for input thermal distribution. Red, no cross-Kerr; blue, with cross-Kerr. Green, output in mode 4^f^.

At the output of the 50/50 merger, modes 2${}^{\prime \prime }$ and 3${}^{\prime \prime }$ become correlated. Their correlation encodes (samples) *n*_ of modes 1 and 4. Consequently, in the weak copies, the *n*_ distribution broadens (increases its entropy), while the ϕ distribution is still uniform (Fig. 2A).

(ii) At the nonlinear feedforward stage, two cross-Kerr couplers cause, in the classical approximation (*59*), the phase difference of the hot modes 1${}^{\prime \prime }$ and 4${}^{\prime \prime }$ to be shifted proportionally to the intensity difference of modes 2${}^{\prime \prime }$ and 3${}^{\prime \prime }$, respectively, so that ϕ → ϕ + χ*n*_${}^{\prime \prime }$, where χ is the nonlinear phase shift. For each set of coherent-state amplitudes in the thermal input distribution, {∣α_1_〉, ∣0_2_〉, ∣0_3_〉, ∣α_4_*e^iϕ^*〉}, the coherent states in the strong-fraction hot modes 1${}^{\prime \prime \prime }$ and 4${}^{\prime \prime \prime }$ then become, after the cross-Kerr couplers (in this classical approximation; see the Supplementary Materials), $|\alpha_{1}^{\prime \prime \prime }\rangle =|c\alpha_{1}\exp\{i\chi \frac{s^{2}}{2}{|\alpha_{1}+\alpha_{4}e^{i\phi }|}^{2}\}\rangle$, $|\alpha_{4}^{\prime \prime \prime }\rangle =|c\alpha_{4}\exp\{i[\chi \frac{s^{2}}{2}{|\alpha_{1}-\alpha_{4}e^{i\phi }|}^{2}+\phi ]\}\rangle$.

We infer the MI from the (*n*_−_ − ϕ) distribution of the modes 1${}^{\prime \prime \prime }$ and 4${}^{\prime \prime \prime }$ (Fig. 2B; see the Supplementary Materials) (*60*). The MI at the nonlinear coupling stage (*k* = 2 in Eq. 1) is diminished compared to that of the sampling stage (*k* = 1), i.e., *I_k_* is reduced since the entropy 𝒮*_k_*(ϕ) reduces, while 𝒮*_k_*(*n*_) is unchanged. This means that the cross-Kerr coupling feeds forward the information encoded by ϕ, resulting in a sharply peaked (narrowed-down) ϕ distribution (Fig. 2C).

(iii) At the steering stage, the final 50/50 BS, preceded by a π/2 shift of mode 4${}^{\prime \prime \prime }$, yields at the two outputs the coherent-state amplitudes $\alpha_{1,4}^{f}=2^{-1/2}(\alpha_{1}^{\prime \prime \prime }\pm i\alpha^{\prime \prime \prime })$, which determine the output intensities

$${|\alpha_{1,4}^{f}|}^{2}=\frac{c^{2}}{2}[\alpha_{1}^{2}+\alpha_{4}^{2}\pm 2\alpha_{1}\alpha_{4}\sin(2s^{2}\alpha_{1}\alpha_{4}\chi \cos\phi -\phi )]$$ (2)

This nonsinusoidal dependence of the interference term on the phase difference ϕ of the input fields stems from the nonlinear coupling. The interference term vanishes in the linear limit χ = 0 upon averaging over ϕ, since the distribution ${\tilde{p}}^{f}(\phi )$ is then flat. It is due to cross-Kerr nonlinearity that the final narrow-peaked ${\tilde{p}}^{f}(\phi )$ allows, for appropriate χ and *s*, to achieve a predominantly destructive interference in mode 4*^f^* and a constructive interference in mode 1*^f^* and thereby net steering of mean intensity from mode 4 to mode 1 (*61*) (or conversely) upon averaging over the random input amplitudes and phase differences ϕ in the thermal input distribution, $P(\alpha_{1},\alpha_{4},\phi )=\frac{2}{\pi {\bar{n}}^{2}}\alpha_{1}\alpha_{4}e^{-\frac{\alpha_{1}^{2}+\alpha_{4}^{2}}{\bar{N}}}$ (Fig. 2D).

The mean intensities at the output of the final 50/50 BS in the strong-fraction hot modes are found to be, in this classical approximation

$${\bar{n}}_{1,4}^{f}=c^{2}\bar{n}\left[1\pm \frac{s^{2}\chi \bar{n}}{{\left(1+s^{4}\chi^{2}{\bar{n}}^{2}\right)}^{2}}\right]$$ (3)

For equal input temperatures without cross-Kerr coupling (χ = 0), there is no steering.

**Fig. 3.**
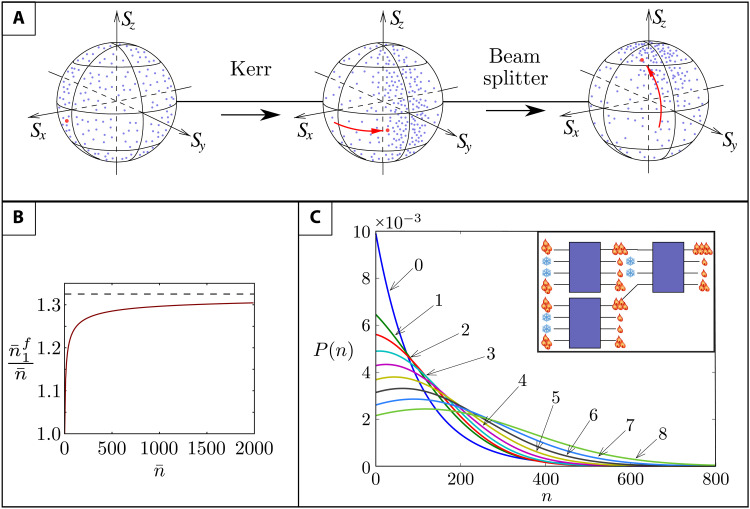
Poincaré sphere representation, output quanta number, and its distribution under casacding for HENLI. Blue dots, coherent states initially distributed randomly on the Poincaré sphere. Red point, a randomly chosen state; red arrows, its transformations. (**A**) The cross-Kerr interaction concentrates the states in the negative *S_y_* hemisphere, and the final BS rotates the sphere by π/2 around *S_x_* to move the states toward *S_z_* > 0. The distribution is eventually concentrated near the north pole (maximal ${\bar{S}}_{z}$). (**B**) Optimized mean output quanta number ${\bar{n}}_{1}^{f}$ in mode 1*^f^*, normalized to the mean input quanta number $\bar{n}$, plotted versus $\bar{n}$ for thermal states of equal temperature in the hot modes 1 and 4. Full line, fully quantum calculation; broken line, classical approximation. (**C**) Cascading the HENLI blocks: The highest-energy outputs of each block are used as the hot-mode inputs of the next one. Quanta number distribution of the highest-energy outputs in eight consecutive blocks shows growing displacement of the distribution mean, i.e., increasing work capacity. The parameters χ and *s* have been optimized to maximize $\bar{n}-\Delta n$ (i.e., the nonpassivity).

The optimal value of χ, $\chi_{\mathrm{opt}}=\frac{1}{\sqrt{3}\bar{n}s^{2}}$ when inserted in Eq. 3, yields ${\left({\bar{n}}_{1}^{f}\right)}_{\left(\max\right)}=c^{2}\bar{n}\left(1+\frac{9}{16\sqrt{3}}\right)\approx (4/3)c^{2}\bar{n}$ (Fig. 3B). This shows that we should split off as little of the input energy as possible (*s*^2^ ≪ 1) and increase Kerr nonlinearity (χ ≫ 1).

### Quantum correlations

Quantum correlations of HENLI are captured by the two-mode Stokes operators (*62*–*66*), expressed for modes *i* and *j* in terms of their annihilation operators ${\hat{a}}_{i(j)}$ as: ${\hat{J}}_{+}^{\left(ij\right)}={\hat{a}}_{i}^{†}{\hat{a}}_{j},{\hat{J}}_{-}^{\left(ij\right)}={\hat{a}}_{i}{\hat{a}}_{j}^{†};\;{\hat{J}}_{z}^{\left(ij\right)}=\frac{1}{2}({\hat{a}}_{i}^{†}{\hat{a}}_{i}-{\hat{a}}_{j}^{†}{\hat{a}}_{j}),{\hat{J}}_{0}^{\left(ij\right)}=\frac{1}{2}({\hat{a}}_{i}^{†}{\hat{a}}_{i}+{\hat{a}}_{j}^{†}{\hat{a}}_{j})$. These Stokes operators evolve via successive unitary operations: Sampling (sa), cross-Kerr nonlinear (nl), and steering (st), $\hat{U}={\hat{U}}_{\mathrm{st}}{\hat{U}}_{\mathrm{nl}}{\hat{U}}_{\mathrm{sa}}$, where ${\hat{U}}_{\mathrm{sa}}$ and ${\hat{U}}_{\mathrm{st}}$ are LIGO rotations on the four-mode Poincaré sphere, whereas ${\hat{U}}_{\mathrm{nl}}$ is a “twisting” NGO that entangles all modes in a quantum nonlinear fashion (see the Supplementary Materials).

The correlation effects are manifest by the Stokes parameters, the mean values of the Stokes operators, of modes 1 and 4, $S_{k}=\langle {\hat{J}}_{k}^{\left(14\right)}\rangle ,\;k=x,y,z,0$. One can associate any two-mode coherent state with a point (*S_x_*, *S_y_*, *S_z_*) on the surface of a Poincaré sphere of radius *S*_0_. Under thermal-ensemble averaging, the first two BSs (LIGO) rotate the distribution, but the crucial nonlinear stage concentrates the random phase-space points on the surface of the hemisphere *S_y_* < 0 (Fig. 3A). Steering via π/2 phase shifter in mode 4${}^{\prime \prime \prime }$ and the output BS brings the points concentrated on the *S_y_* < 0 hemisphere to the *S_z_* > 0 hemisphere surface. This energy steering does not violate the Liouville theorem, because the contraction of the phase-space volume of modes 1 and 4 is compensated by the expansion of the volume of modes 2 and 3.

When the input modes 1 and 4 are coherent states with equal amplitudes, α_1_ = α_4_, and random phases, the average quanta numbers of the output modes are found to be (see the Supplementary Materials)

$$\bar{\left\langle {\hat{n}}_{1,4}^{\left(f\right)}\right\rangle }={\bar{S}}_{0}^{\left(f\right)}\pm {\bar{S}}_{z}^{\left(f\right)}=c^{2}\alpha_{1}^{2}[1\pm J_{1}(b)e^{-d}]$$ (4)

where ${\hat{n}}_{i}={\hat{a}}_{i}^{†}{\hat{a}}_{i}$, $d=s^{2}(1-\cos\chi )(\alpha_{1}^{2}+\alpha_{4}^{2})$, *J*_1_(*b*) is the first-order Bessel function with argument *b* = 2*s*^2^α_1_α_4_sinχ, and the overbar denotes averaging over random phase difference ϕ. When the fields are classical, the average field intensities in Eq. 4 have *d* = 0 and *b* = 2*s*^2^α_1_α_4_χ (see the Supplementary Materials). Nonzero *d* value results from vacuum fluctuations of the modes and gives rise to exponential decay that diminishes the energy steering in Eq. 4 as compared to Eq. 3 in the classical approximation (Fig. 3B). This represents quantum disadvantage compared to classical HENLI.

### Cascading

One can concentrate the energy to higher values by cascading the four-mode blocks described above (Fig. 3C, inset). Such a cascade of blocks can be viewed as the spatial analog of consecutive temporal strokes of an HE. In each block, the relative variance $\Delta n/\bar{n}$ becomes smaller than in the preceding one. This cascading (Fig. 3) yields an increasingly nonmonotonic quanta number distribution {*p_n_*}(*n* = 0,1,2...), i.e., increasingly nonpassive state in the working mode.

Although analytical formulae are tractable only for the first two moments of the distribution, we can put a bound on its nonmonotonicity by choosing the distribution with the highest entropy that corresponds to the values of the first two moments, consistent with the Jaynes principle (*67*).

The input energy fraction converted to work is well below the Carnot bound. The ability to attain this bound by cascading is yet to be studied and so is the steady state of such a cascade.

## DISCUSSION

### Implementations

**Fig. 4.**
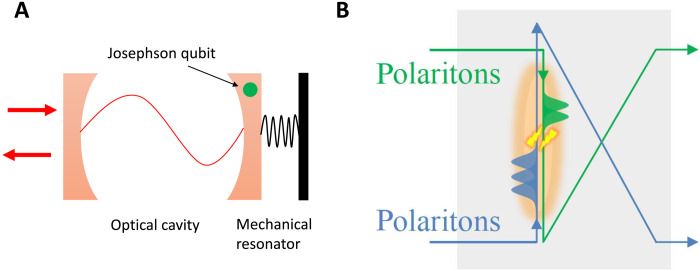
Experimental implementations. (**A**) Optomechanical implementation: Optical cavity and mechanical resonator cross-Kerr coupled by a Josephson qubit (*69*). (**B**) Quantum optical implementation: Photons from two input modes counterpropagate as slow-light Rydberg polaritons whose dipole-dipole interaction leads to a cross-Kerr phase shift.

Among conceivable cross-Kerr mechanisms for few quanta (*68*), two are clearly feasible: (i) In an optomechanical setup, a microwave cavity can be coupled to a mechanical resonator by a Josephson qubit (Fig. 4A) that induces cross-Kerr coupling between the cavity and the resonator (*69*, *70*). The cavity-field phase shift will strongly depend on the phonon number in the mechanical resonator as χ per phonon can be large (*69*).

(ii) Slow-light Rydberg polaritons can yield giant cross-Kerr nonlinearity according to our theoretical ideas (*71*, *72*) and experiments (*73*–*75*) with few-photon beams converted into cold-atom Rydberg polaritons (Fig. 4B). Their dipolar interaction can yield a phase shift of χ ≃ π between each pair of counterpropagating photons.

Nonlinear interferometric networks have been proposed as unconventional, fully coherent, closed-system, autonomous HEs. The analysis of the minimal HENLI four-mode cross-Kerr coupled network shows that quantum correlations of the coupled modes incur vacuum noise, which is a disadvantage compared to classical correlations.

HENLI may give rise to new technologies of steering ambient heat (few-quanta input) in multimode networks and its conversion to quasi-coherent work output: (i) The cascading process (Fig. 3C) may allow manipulating and enhancing the information hidden in noisy input via controllable nonlinear operations, which bears HENLI’s remarkable resemblance to a quantum computer with continuous variables (*76*), if intermode quantum correlations are accounted for, or to a classical optical computer, if they are neglected. (ii) The strongly reduced phase and amplitude uncertainty of the non-Gaussian output obtained by the nonlinear transformation of the thermal output can be used for quantum sensing and phase estimation (*77*, *78*). (iii) The feasibility of cross-Kerr coupling for few-photon or few-phonon input (Fig. 4) may facilitate the creation of nonlinear interference devices (*71*–*76*) for quantum logic or communications. (iii) Sophisticated versions of HENLI (to be reported) with up to eight modes in each block may realize functionalities other than HE [e.g., refrigerator (*35*), heat transistor (*37*–*39*), or sensors of noise cross-correlations].

By replacing open systems with closed systems, our long-term goal is to trace the transition from quantum or classical coherent behavior to thermodynamics as a function of the number of modes and their nonlinear coupling. Such a transition may lay the ground for bridging the conceptual gulf between nonlinear dynamics and thermodynamics.

## METHODS

### HENLI principles

Consider a multiport linear interferometer with *m* input modes and *m* output modes that contains only (energy-conserving and therefore passive) linear mode couplers or BSs. If a multimode factorized coherent state ∣β_1_〉 ∣ β_2_〉… ∣ β*_m_*〉 is the input, then one can find parameters of the interferometer that give rise to a coherent state ∣α〉 in one output mode, all the remaining output modes being empty, i.e., full energy concentration is achievable.

If, however, the input is thermal noise, which can be treated as a distribution of coherent states ∣β_1_〉 ∣ β_2_〉… ∣ β*_m_*〉 with random amplitudes of β_1_, β_2_, …β*_m_* that have Gaussian distributions with zero mean, then neither of the HE functionalities is then achievable via linear interferometric network (*41*, *42*), as the thermal randomness prevents selected-mode amplification or heat-to-work conversion (*43*, *44*).

However, if we could estimate the magnitudes and phases of β_1_, β_2_, …β*_m_* and feedforward the results for each realization of the random input, then we would be able to choose the interferometer parameters such that the energy is mostly concentrated in nonpassive form in one mode. Instead of conventional measurements that can provide this information nearly perfectly (*33*), we show that it is possible to partially estimate and feedforward them autonomously by nonlinear intermode coupling, which is inevitably NGO. This feat cannot be achieved even by NGO if all the input modes are in the same thermal state, as follows from the second law of thermodynamics: Some of the inputs have to be colder than others, forming distinct cold and hot few-mode baths.

The general *m*-mode HENLI protocol consists of two major stages (Fig. 1A):

1) Sampling: A fraction of each hot input field mode is split off and mixed with a corresponding cold field mode by imbalanced BS. The cold-mode states then become weak copies (perfect copies, assuming that the cold modes are empty) of the respective hot-mode states. These *m*/2 weak copies are pairwise mixed by 50/50 merger. These LIGOs “sample” the random distribution of the input modes: The “sampled” phase differences are encoded by the intensity mixing ratio of the weak-copy outputs.

2) Nonlinear feedforward: Subsequently, these weak-copy outputs are nonlinearly cross-correlated by NGO (here, nonlinear cross-Kerr coupling) with the dominant hot-mode fractions. Since the cross-Kerr Hamiltonian commutes with the bare Hamiltonian of the hot and cold modes, it does not require energy investment, nor does it require external control, so that HENLI is a self-contained (autonomous) heat-to-work converter. An additional *m*-mode basis rotation and phase shifting exploit this autonomous nonlinear feedforward of the sampling to steer the energy mainly to the desired mode. The output distributions become nonthermal (non-Gaussian).

## Supplementary Materials

### This PDF file includes:

Sections SI to SIII
