# Supplementary material for: Nonlinear coherent heat machines
Source: Sci Adv. 2023 Jan 6;9(1):eadf1070. doi: 10.1126/sciadv.adf1070 (PMC9821940; doi:10.1126/sciadv.adf1070)
Supplement: Supplementary file 1 — Sections SI to SIII [file sciadv.adf1070_sm.pdf]

Science Advances
AAAS

Supplementary Materials for

Nonlinear coherent heat machines

Tomáš Opatrný *et al.*

Corresponding author: Tomáš Opatrný, tomas.opatrny@upol.cz; Avijit Misra, avijitmisra0120@gmail.com; Nilakantha Meher, nilakantha.meher6@gmail.com

Sci. Adv. **9**, eadf1070 (2023)
DOI: 10.1126/sciadv.adf1070

This PDF file includes:

Sections SI to SIII

I. HENLI OUTPUT INTENSITIES WITH MEAN INTER-MODE CORRELATIONS

In this section we resort to the coherent state basis, which underscores the quantum-classical analogy, and average the input over random phases or thermal distributions. The cross-Kerr mode correlations are replaced by their classical (mean) values, so that the intermode quantum entanglement and quantum fluctuations are here neglected. The full 4-mode quantum correlations are treated in Sec. III below. The approximation in this section is denoted as "classical" in the main text.

The Fock state representation of a two-mode coherent state with amplitudes α and β is given by

$$|\alpha\rangle|\beta\rangle = e^{-\frac{|\alpha|^2}{2}} e^{-\frac{|\beta|^2}{2}} \sum_{m,n} \frac{\alpha^n}{\sqrt{n!}} \frac{\beta^m}{\sqrt{m!}} |n\rangle|m\rangle. \tag{1}$$

The cross-Kerr interaction transforms Fock states as $|n\rangle|m\rangle \rightarrow e^{i\chi nm}|n\rangle|m\rangle$, so that the input two-mode coherent state changes to the two-mode entangled state

$$U_{\mathrm{cK}}|\alpha\rangle|\beta\rangle = e^{-\frac{|\alpha|^2}{2}} e^{-\frac{|\beta|^2}{2}} \sum_{m,n} \frac{\alpha^n}{\sqrt{n!}} \frac{\beta^m}{\sqrt{m!}} e^{i\chi nm} |n\rangle|m\rangle. \tag{2}$$

The density matrix corresponding to this state is

$$\rho = e^{-|\alpha|^2} e^{-|\beta|^2} \sum_{n,m,n',m'} \frac{\alpha^n \alpha^{*n'} \beta^m \beta^{*m'}}{\sqrt{n!n'!m!m'!}} e^{i\chi(nm-n'm')} |n,m\rangle\langle n',m'|. \tag{3}$$

The reduced density matrix related to the mode with coherent-state amplitude α is then

$$\begin{aligned} \rho_\alpha = \mathrm{Tr}_\beta \rho &= e^{-|\alpha|^2} e^{-|\beta|^2} \sum_{n,m,n'} \frac{\alpha^n \alpha^{*n'} |\beta|^{2m}}{\sqrt{n!n'!}\ m!} e^{i\chi(n-n')m} |n\rangle\langle n'| \\ &= e^{-|\alpha|^2} \sum_{n,n'} \frac{\alpha^n \alpha^{*n'}}{\sqrt{n!n'!}} \exp\left\{|\beta|^2 \left[e^{i\chi(n-n')} - 1\right]\right\} |n\rangle\langle n'|. \end{aligned} \tag{4}$$

This result enables us to find moments of the creation and annihilation operators acting on mode α. The first moment is

$$\begin{aligned} \langle \hat{a} \rangle &= \mathrm{Tr}[\hat{a}\rho_\alpha] = e^{-|\alpha|^2} \mathrm{Tr} \sum_{n,n'} \frac{\alpha^n \alpha^{*n'}}{\sqrt{n!n'!}} \exp\left\{|\beta|^2 \left[e^{i\chi(n-n')} - 1\right]\right\} \sqrt{n}|n-1\rangle\langle n'| \\ &= e^{-|\alpha|^2} \mathrm{Tr} \sum_{k,n'} \frac{\alpha^{k+1} \alpha^{*n'}}{\sqrt{(k+1)!n'!}} \exp\left\{|\beta|^2 \left[e^{i\chi(k+1-n')} - 1\right]\right\} \sqrt{k+1}|k\rangle\langle n'| \\ &= e^{-|\alpha|^2} \sum_{k} \frac{\alpha^{k+1} \alpha^{*k}}{k!} \exp\left[|\beta|^2 \left(e^{i\chi} - 1\right)\right] \\ &= \alpha \exp\left[|\beta|^2 \left(e^{i\chi} - 1\right)\right]. \end{aligned} \tag{5}$$

In the same way one can find mean values of normally-ordered higher-order two-mode correlations:

$$\langle \hat{a}^{\dagger q} \hat{a}^r \rangle = \alpha^{*q} \alpha^r \exp\left[|\beta|^2 \left(e^{i\chi(r-q)} - 1\right)\right], \tag{6}$$

where q, r are positive integers.

Now consider the input $|\alpha_1\rangle\,|0_2\rangle\,|0_3\rangle\,|\alpha_4 e^{i\phi}\rangle$ to the 4-mode HENLI (Fig. 4(b) in main text). All the figure numbers mentioned in this supplementary material correspond to the figures in the main text. The states after the sampling stage (after the first 50/50 BS in Fig. 4b) are

$$|\alpha_1''\rangle = |c\alpha_1\rangle, \quad |\alpha_4''\rangle = |c\alpha_4 e^{i\phi}\rangle \tag{7}$$

$$|\alpha_{2(3)}''\rangle = |\frac{s}{\sqrt{2}}\left(\alpha_1 \pm \alpha_4 e^{i\phi}\right)\rangle, \tag{8}$$

It follows from these expressions that, in the case of equal input in modes 1 and 4, equal mixing of their weak copies by this LIGO occurs for $|\phi| = \pi/2$, while more energy flows towards $2''$ for $|\phi| \leq \pi/2$ (100% towards $2''$ for $\phi = 0$) and conversely for $\pi \geq |\phi| > \pi/2$.

The annihilation operators of modes $1'''$ and $2'''$ after the cross-Kerr coupling are $\hat{a}_1'''$ and $\hat{a}_4'''$ with mean values

$$\begin{aligned} \langle \hat{a}_1''' \rangle &= c\alpha_1 \exp\left\{ \frac{s^2}{2} \left[\left| \alpha_1 + \alpha_4 e^{i\phi} \right|^2 \left(e^{i\chi} - 1 \right) \right] \right\}, \\ \langle \hat{a}_4''' \rangle &= c\alpha_4 \exp\left\{ \frac{s^2}{2} \left[\left| \alpha_1 - \alpha_4 e^{i\phi} \right|^2 \left(e^{i\chi} - 1 \right) \right] + i\phi \right\}. \end{aligned} \tag{9}$$

The output fields following the phase shifter in mode $4'''$ and the final BS have the annihilation operators

$$\begin{aligned} \hat{a}_1^f &= \frac{1}{\sqrt{2}} \left(\hat{a}_1''' + \hat{a}_4''' e^{i\frac{\pi}{2}} \right), \\ \hat{a}_4^f &= \frac{1}{\sqrt{2}} \left(\hat{a}_1''' - \hat{a}_4''' e^{i\frac{\pi}{2}} \right), \end{aligned} \tag{10}$$

with the mean output quantum numbers

$$\langle \hat{n}_{1,4}^f \rangle = \frac{c^2}{2} \{ \alpha_1^2 + \alpha_4^2 \pm 2\alpha_1\alpha_4 \exp\left[-2s^2(\alpha_1^2 + \alpha_4^2)\sin^2\frac{\chi}{2} \right] \sin\left(2s^2\alpha_1\alpha_4 \sin\chi \cos\phi - \phi \right) \}. \tag{11}$$

Here $\chi \neq 0$ leads to nonlinear and non-sinusoidal dependence on ϕ, α_1 and α_4.

We now proceed to average the above results over the input distributions.

A. Coherent-state input with random phases

For the mean quantum numbers at the outputs 1^f and 4^f, we have

$$\bar{n}_{1,4}^f \equiv \int_0^\infty \int_0^\infty \int_0^{2\pi} \int_0^{2\pi} \langle \hat{n}_{1,4}^f \rangle P(\alpha_1, \psi, \alpha_4, \psi + \phi) d\phi \, d\psi \, d\alpha_1 \, d\alpha_4. \tag{12}$$

This result is valid for states which can be constructed as mixtures of coherent states, i.e., those with *regular Glauber-Sudarshan distribution.*

The Glauber-Sudarshan function of the input state is taken here to have fixed equal amplitudes but random phases

$$P(\alpha_1, \psi, \alpha_4, \psi + \phi) = \frac{1}{(2\pi)^2} \delta(\alpha_1 - \sqrt{\bar{n}}) \delta(\alpha_4 - \sqrt{\bar{n}}). \tag{13}$$

From Eq. (12), we then get

$$\bar{n}_{1,4}^f = c^2 \bar{n} \pm G \tag{14}$$

with

$$G = c^2 \bar{n} \exp\left(-4s^2 \bar{n} \sin^2\frac{\chi}{2} \right) J_1\left(2s^2 \bar{n} \sin\chi \right). \tag{15}$$

In this subsection, we adopt the classical approximation for coherent state input. For two coherent field modes with equal amplitudes $\alpha_1 = \alpha_4 = \sqrt{\bar{n}}$ and relative phase ϕ distributed with uniform probability density in the 2π interval, Eq. (4) in the main text, averaged over ϕ, becomes

$$\bar{n}_{1,4}^f = c^2 \bar{n} \left[1 \pm J_1\left(2s^2 \chi \bar{n} \right) \right], \tag{16}$$

where $\bar{n}_i^f = |\alpha_i^f|^2$ is the output intensity of mode i, $J_1(x)$ is the appropriate Bessel function of the first kind. The interference term has a maximum for $2s^2\chi\bar{n} \approx 1.84$ where the Bessel function reaches ≈ 0.58, indicating that about 58% of the energy of mode 4 can be transferred to mode 1. This occurs in the limit of $c^2 \to 1$, i.e., $s^2 \to 0$ and $\chi\bar{n} \to \infty$.

For two coherent input modes with unequal amplitudes, the question is what fraction of the weaker field can be transferred to the stronger one. For this purpose it is useful to choose the input BSs to have different splitting ratios such that $s_1^2\bar{n}_1 = s_2^2\bar{n}_4$, so that the split-off weak fractions have equal intensities when they interfere before entering the cross-Kerr couplers. We then optimize the splitting ratio of the final (output-f) BS, denoting its parameters as c_f and s_f. The output intensities are then

$$\bar{n}_{1,4}^f = c_f^2 c_1^2 \bar{n}_1 + s_f^2 c_2^2 \bar{n}_4 \pm 2 c_f s_f c_1 c_2 \sqrt{\bar{n}_1 \bar{n}_4} J_1\left(2 s_1 s_2 \chi \sqrt{\bar{n}_1 \bar{n}_4}\right). \tag{17}$$

The optimized energy transfer in the regime of strong nonlinearity and weak splitting off, $s_{1,2} \to 0$, $\chi \to \infty$ requires that $2 s_1 s_2 \chi \sqrt{\bar{n}_1 \bar{n}_4} \approx 1.84$ where the Bessel function reaches its maximum. The optimized energy in mode 1^f is then found to be

$$\bar{n}_1^f = \frac{2\bar{n}_1\bar{n}_4 J_M^2\left[\bar{n}_1 + \bar{n}_4 + \sqrt{4\bar{n}_1\bar{n}_4 J_M^2 + 2(\bar{n}_1 - \bar{n}_4)^2}\right] + \bar{n}_1(\bar{n}_1 - \bar{n}_4)^2}{4\bar{n}_1\bar{n}_4 J_M^2 + (\bar{n}_1 - \bar{n}_4)^2}, \tag{18}$$

where $J_M \approx 0.58$ is the maximum value of the Bessel function. In the limit of $\bar{n}_4 \ll \bar{n}_1$, $\bar{n}_{1,4}^f \approx \bar{n}_1 + 0.28\bar{n}_4$, i.e., up to about 28% of the weak-field energy can be transferred to the higher energy mode.

Although there is virtually zero energy transfer to the cold modes and the energy on average climbs uphill to the higher energy mode, there is *no violation of the second law*: we start from fields with non-fluctuating amplitudes and end up with fields whose amplitudes fluctuate according to their random mutual input phases.

B. Thermal-state inputs with equal temperature

For a thermal state with mean quantum number $\bar{n}$ the single mode probability distribution is

$$P(\alpha, \phi) = \frac{1}{\pi\bar{n}} \alpha e^{-\alpha^2/\bar{n}}. \tag{19}$$

Assuming that both of the hot modes (1,4) are at the same temperature with mean quantum number $\bar{n}$, the integrals in Eq. (12) lead to

$$\bar{n}_1^f = c^2\bar{n} + G, \tag{20}$$

$$\bar{n}_4^f = c^2\bar{n} - G, \tag{21}$$

where the "gain" term is

$$\begin{aligned} G &= c^2 \int_0^\infty \int_0^\infty \int_0^{2\pi} \int_0^{2\pi} a_1 a_4 e^{-2s^2(a_1^2+a_4^2)\sin^2\frac{\chi}{2}} \sin\left(2s^2 a_1 a_4 \sin\chi\cos\phi - \phi\right) \frac{1}{(\pi\bar{n})^2} a_1 a_4 e^{-\frac{a_1^2+a_4^2}{\bar{n}}} d\phi d\psi da_1 da_4 \\ &= \frac{4c^2}{\bar{n}^2} \int_0^\infty \int_0^\infty x^2 y^2 \exp\left[-\left(\frac{1}{\bar{n}} + 2s^2\sin^2\frac{\chi}{2}\right)(x^2+y^2)\right] J_1\left(2s^2\sin\chi xy\right) dxdy. \end{aligned} \tag{22}$$

The integrals can be evaluated as follows. Substituting

$$a \equiv \frac{1}{\bar{n}} + 2s^2\sin^2\frac{\chi}{2}, \tag{23}$$

$$b \equiv 2s^2\sin\chi, \tag{24}$$

$$x = r\cos\xi, \tag{25}$$

$$y = r\sin\xi, \tag{26}$$

$$dx\, dy = rdr\, d\xi, \tag{27}$$

we get

$$G = \frac{4c^2}{\bar{n}^2} \int_0^{\pi/2} \cos^2\xi \sin^2\xi \int_0^\infty r^5 \exp\left(-ar^2\right) J_1\left(b\sin\xi\cos\xi\, r^2\right) dr\, d\xi \tag{28}$$

$$= \frac{2c^2}{\bar{n}^2} \int_0^{\pi/2} \cos^2\xi \sin^2\xi \int_0^\infty u^2 \exp\left(-au\right) J_1\left(b\sin\xi\cos\xi\, u\right) du\, d\xi. \tag{29}$$

Integral over u gives

$$G = \frac{6c^2ab}{\bar{n}^2}\int_0^{\pi/2}\frac{\sin^3\xi\cos^3\xi}{\left(a^2+b^2\sin^2\xi\cos^2\xi\right)^{5/2}}d\xi, \tag{30}$$

and integrating the result over ξ we get

$$G = \frac{c^2s^2\sin\chi}{\bar{n}^2\left[\left(\frac{1}{\bar{n}}+2s^2\sin^2\frac{\chi}{2}\right)^2+s^4\sin^2\chi\right]^2}. \tag{31}$$

C. Ergotropy calculation

The output distributions in modes 1^f and 4^f are generally non-thermal. They become increasingly non-passive when cascaded through consecutive blocks (Fig. 2c). The ergotropy of such modes can be calculated using

$$\mathcal{W} = \sum_n n(\rho_n - \rho_n^{(pas)}), \tag{32}$$

where $\{\rho_n^{(pas)}\}$ are the passive states of quantum numbers n having monotonically decreasing probabilities. These probabilities are obtained by permuting the state probabilities, so that they become monotonically decreasing.

II. PHASE-INTENSITY MUTUAL INFORMATION IN HENLI

The conditional probability distribution of the quantum number difference n_- of the weak-copy modes 2 and 3 after the first 50/50 BS (sampling stage) is, in the Gaussian approximation

$$p_1(n_-|\alpha_1,\alpha_4,\phi) = \frac{1}{\sqrt{2\pi}s\sqrt{\alpha_1^2+\alpha_4^2}}\exp\left[-\frac{(n_- - 2s^2\alpha_1\alpha_4\cos\phi)^2}{2s^2(\alpha_1^2+\alpha_4^2)}\right]. \tag{33}$$

One can get the conditional probability $p(n_-|\phi)$ by averaging over α_1 and α_4. Assuming thermal distributions for both of them

$$p(\alpha_1) = \frac{2}{\bar{n}_T}\alpha_1 e^{-\frac{\alpha_1^2}{\bar{n}_T}}, \tag{34}$$

$$p(\alpha_4) = \frac{2}{\bar{n}_T}\alpha_4 e^{-\frac{\alpha_4^2}{\bar{n}_T}}, \tag{35}$$

one finds

$$p_1(n_-|\phi) = \frac{4}{\sqrt{2\pi}s\bar{n}_T^2}\int_0^\infty\int_0^\infty\frac{\alpha_1\alpha_4}{\sqrt{\alpha_1^2+\alpha_4^2}}\exp\left[-\frac{\alpha_1^2+\alpha_4^2}{\bar{n}_T}-\frac{(n_- - 2s^2\alpha_1\alpha_4\cos\phi)^2}{2s^2(\alpha_1^2+\alpha_4^2)}\right]d\alpha_1 d\alpha_4 \tag{36}$$

$$= \frac{1}{\sqrt{2\pi}s\bar{n}_T^2}\int_0^\infty\int_0^\pi\exp\left[-\frac{r^2}{\bar{n}_T}-\frac{(n_- - s^2r^2\sin\xi\cos\phi)^2}{2s^2r^2}\right]r^2\sin\xi d\xi dr, \tag{37}$$

where is the last integral we used the substitution $\alpha_1 \equiv r\cos\frac{\xi}{2}$ and $\alpha_4 \equiv r\sin\frac{\xi}{2}$.

The joint probability distribution $\mathcal{P}_1(n_-,\phi)$ is obtained by multiplying

$$\mathcal{P}_1(n_-,\phi) = p_1(n_-|\phi)\tilde{p}(\phi), \tag{38}$$

where $\tilde{p}(\phi) = 1/(2\pi)$ is constant.

These quantities can be used to find the mutual information between n_- and the quantities α_1, α_4 and ϕ,

$$I_{n_-;\alpha_1,\alpha_4,\phi} = \int\int\int\int p_1(n_-|\alpha_1,\alpha_4,\phi)p(\alpha_1)p(\alpha_4)p(\phi)\ln\frac{p_1(n_-|\alpha_1,\alpha_4,\phi)}{p_1(n_-)}dn_- d\alpha_1 d\alpha_4 d\phi, \tag{39}$$

where

$$p_1(n_-) = \int \mathcal{P}_1(n_-, \phi) d\phi. \tag{40}$$

As an example we used $\bar{n}_T = 50$, $s^2 = 0.1$ to find $I_{n_-;\alpha_1,\alpha_4,\phi} \approx 0.97$.

Disregarding the magnitudes α_1 and α_4 one can find the mutual information of n_- and ϕ as

$$I_{n_-;\phi} = \int\int p_1(n_-|\phi) p(\phi) \ln \frac{p_1(n_-|\phi)}{p_1(n_-)} dn_- d\phi. \tag{41}$$

Taking the same parameters as in the preceding case we get $I_{n_-;\phi} \approx 0.48$.

Consistently with the approximation in Sec. I, the modes 1 and 4 are then subject to a cross-Kerr phase shift proportional to n_-, so the coherent states can be written as $|\alpha_1\rangle$, $|\alpha_4 e^{i(\phi+\chi n_-)}\rangle$. After states are combined on the final 50/50 BS with additional phase shift $\pi/2$, the outputs have approximately Gaussian distributions with means and variances

$$\bar{n}_\pm = \sigma^2_{n,\pm} = \frac{c^2}{2}\left[\alpha_1^2 + \alpha_4^2 \pm 2\alpha_1\alpha_4 \sin(\phi + \chi n_-)\right], \tag{42}$$

i.e.,

$$p_2(n|\alpha_1, \alpha_4, \phi, n_-) = \frac{1}{\sqrt{2\pi} c \sqrt{\frac{\alpha_1^2+\alpha_4^2}{2} \pm 2\alpha_1\alpha_4 \sin(\phi + \chi n_-)}} \exp\left\{ -\frac{c^2\left[\frac{n}{c^2} - \frac{\alpha_1^2+\alpha_4^2}{2} \mp \alpha_1\alpha_4 \sin(\phi + \chi n_{--})\right]^2}{2\left[\frac{\alpha_1^2+\alpha_4^2}{2} \pm \alpha_1\alpha_4 \sin(\phi + \chi n_{--})\right]} \right\}. \tag{43}$$

Then

$$p_2 \ (n|\alpha_1, \alpha_4, \phi, n_-) = \frac{1}{\sqrt{2\pi} c \sqrt{\frac{\alpha_1^2+\alpha_4^2}{2} \pm 2\alpha_1\alpha_2 \sin(\phi+\chi n_-)}} \tag{44}$$

$$\exp\left[-\frac{c^2[\frac{n}{c^2} - (\frac{\alpha_1^2+\alpha_4^2}{2} \pm 2\alpha_1\alpha_2 \sin(\phi+\chi n_-))]^2}{2[\frac{\alpha_1^2+\alpha_4^2}{2} \pm 2\alpha_1\alpha_2 \sin(\phi+\chi n_-)]} \right].$$

One can find the dependence $p(n|\phi)$ by integration

$$p_2(n|\phi) = \int\int\int p(n|\alpha_1, \alpha_4, \phi, n_-) p(\alpha_1, \alpha_4, \phi, n_-) d\alpha_1 d\alpha_4 dn_- \tag{45}$$

$$= \int\int\int p(n|\alpha_1, \alpha_4, \phi, n_-) p(n_-|\alpha_1, \alpha_4, \phi) p(\alpha_1) p(\alpha_4) d\alpha_1 d\alpha_4 dn_-. \tag{46}$$

We thus get

$$p_2(n|\phi) = \frac{2\sqrt{2}}{\pi c s \bar{n}_T^2} \int_{-\infty}^{\infty}\int_0^{\infty}\int_0^{\infty} \frac{\alpha_1\alpha_4}{\sqrt{\alpha_1^2+\alpha_4^2}\sqrt{\alpha_1^2+\alpha_4^2 \pm 2\alpha_1\alpha_4 \sin(\phi+\chi n_-)}}$$

$$\times \exp\left\{ -\frac{\alpha_1^2+\alpha_4^2}{\bar{n}_T} - \frac{(n_- - 2s^2\alpha_1\alpha_4\cos\phi)^2}{2s^2(\alpha_1^2+\alpha_4^2)} - \frac{c^2\left[\frac{n}{c^2} - \frac{\alpha_1^2+\alpha_4^2}{2} \mp \alpha_1\alpha_4\sin(\phi+\chi n_{--})\right]^2}{2\left[\frac{\alpha_1^2+\alpha_4^2}{2} \pm \alpha_1\alpha_4 \sin(\phi+\chi n_{--})\right]} \right\} d\alpha_1 d\alpha_4 dn_- \tag{47}$$

$$= \frac{\sqrt{2}}{2\pi c s \bar{n}_T^2} \int_{-\infty}^{\infty}\int_0^{\infty}\int_0^{\pi} \frac{r\sin\xi}{\sqrt{1 \pm \sin\xi \sin(\phi+\chi n_-)}}$$

$$\exp\left\{ -\frac{r^2}{\bar{n}_T} - \frac{(n_- - s^2r^2\sin\xi\cos\phi)^2}{2s^2r^2} - \frac{c^2\left[\frac{n}{c^2} - \frac{r^2}{2}(1 \pm \sin\xi\sin(\phi+\chi n_-))\right]^2}{r^2(1 \pm \sin\xi\sin(\phi+\chi n_-))} \right\} d\xi dr dn_-. \tag{48}$$

These results are shown in Fig. 1.

By integrating over ϕ one gets the probability distribution of n, i.e.,

$$p_2(n) = \frac{1}{2\pi}\int_0^{2\pi} p_2(n|\phi) d\phi. \tag{49}$$

These results are shown in Fig. 1.

III. QUANTUM CORRELATIONS AND STOKES PARAMETERS

A. Mode operators

For the 4-mode HENLI the unitary evolution operator is

$$\hat{U} = \hat{U}_{\rm S}\hat{U}_{\rm NL}\hat{U}_{\rm M}. \tag{50}$$

Here we used the definitions:

$$\begin{aligned}
&\hat{U}_{\rm M} = \hat{U}_{\rm bs}^{(23)}(\pi/4)\hat{U}_{\rm bs}^{(12)}(\theta)\hat{U}_{\rm bs}^{(34)}(\theta),\\
&\hat{U}_{\rm NL} = \hat{U}_{\rm cK}^{(12)}\hat{U}_{\rm cK}^{(34)}; \;\; \hat{U}_{\rm cK}^{(ij)} = e^{i\chi\hat{n}_i\hat{n}_j},\\
&\hat{U}_{\rm S} = \hat{U}_{\rm bs}^{(14)}(\theta')e^{-i\gamma\hat{n}_4},
\end{aligned} \tag{51}$$

where $\hat{U}_{\rm bs}$ is a two-mode BS-mixing operator and $\hat{U}_{\rm cK}$ is the two-mode cross-Kerr nonlinear operator. The mode operators are transformed by a BS as follows,

$$\begin{pmatrix} \hat{a}_i' \\ \hat{a}_j' \end{pmatrix} = \hat{U}_{\rm bs}^{(ij)\dagger}(\theta)\begin{pmatrix} \hat{a}_i \\ \hat{a}_j \end{pmatrix}\hat{U}_{\rm bs}^{(ij)}(\theta) = B\begin{pmatrix} \hat{a}_i \\ \hat{a}_j \end{pmatrix}. \tag{52}$$

Here

$$B = \begin{pmatrix} c & s \\ -s & c \end{pmatrix}, \tag{53}$$

where $c = \cos\theta$ and $s = \sin\theta$.

The evolution can be recast as a product of the unitary operators corresponding to the elementary operations in HENLI,

$$\hat{U} = \hat{U}_4\hat{U}_3\hat{U}_2\hat{U}_1. \tag{54}$$

Here we used the definitions:

$$\hat{U}_1 = \hat{U}_{\rm bs}^{(12)}(\theta)\hat{U}_{\rm bs}^{(34)}(\theta), \quad \hat{U}_2 = \hat{U}_{\rm bs}^{(23)}(\pi/4), \quad \hat{U}_3 = \hat{U}_{\rm NL} = \hat{U}_{\rm cK}^{(12)}\hat{U}_{\rm cK}^{(34)}, \quad \hat{U}_4 = \hat{U}_{\rm bs}^{(14)}(\pi/4)e^{-i\pi\hat{n}_4/2}. \tag{55}$$

The overall evolution operator

$$\hat{U}_{m,{\rm t}} = \hat{U}_m \ldots \hat{U}_1, \tag{56}$$

transforms the operators $\hat{a}_i$ of the initial modes to the operators $\hat{a}_i^{(m)}$ of the modes obtained after m transformations where m also equals the number of primes denoting the corresponding modes in Fig. 4(b) in the main text,

$$\hat{a}_i^{(m)} = \hat{U}_{m,{\rm t}}^\dagger\hat{a}_i\hat{U}_{m,{\rm t}}. \tag{57}$$

Correspondingly,

$$\hat{U}_m^{(m-1)} = \hat{U}_{m-1,{\rm t}}^\dagger\hat{U}_m\hat{U}_{m-1,{\rm t}} \tag{58}$$

denotes the operator $\hat{U}_m$ expressed through the annihilation and creation operators of the respective primed modes shown in Fig. 4. The evolution operators (56) can be recast in the form,

$$\hat{U}_{m,{\rm t}} = \hat{U}_1 \ldots \hat{U}_m^{(m-1)}. \tag{59}$$

The order of operators in (59) is reversed with respect to (56). This allows us to derive the recursive relation from (57),

$$\hat{a}_i^{(m)} = \hat{U}_m^{(m-1)\dagger}\hat{a}_i^{(m-1)}\hat{U}_m^{(m-1)}. \tag{60}$$

From Eqs. (60), (55), (52), and (53), we obtain that

$$\begin{pmatrix} \hat{a}_1' \\ \hat{a}_2' \\ \hat{a}_3' \\ \hat{a}_4' \end{pmatrix} = \hat{U}_1^\dagger \begin{pmatrix} \hat{a}_1 \\ \hat{a}_2 \\ \hat{a}_3 \\ \hat{a}_4 \end{pmatrix} \hat{U}_1 = \begin{pmatrix} c\hat{a}_1 + s\hat{a}_2 \\ -s\hat{a}_1 + c\hat{a}_2 \\ c\hat{a}_3 + s\hat{a}_4 \\ -s\hat{a}_3 + c\hat{a}_4 \end{pmatrix}, \tag{61}$$

$$\begin{pmatrix} \hat{a}_1'' \\ \hat{a}_2'' \\ \hat{a}_3'' \\ \hat{a}_4'' \end{pmatrix} = \hat{U}_2'^\dagger \begin{pmatrix} \hat{a}_1' \\ \hat{a}_2' \\ \hat{a}_3' \\ \hat{a}_4' \end{pmatrix} \hat{U}_2' = \begin{pmatrix} \hat{a}_1' \\ (\hat{a}_2' + \hat{a}_3')/\sqrt{2} \\ (-\hat{a}_2' + \hat{a}_3')/\sqrt{2} \\ \hat{a}_4' \end{pmatrix}. \tag{62}$$

To obtain the influence of the Kerr effect, we note that

$$\hat{U}_{\mathrm{cK}}^{(ij)\dagger} \hat{a}_i \hat{U}_{\mathrm{cK}}^{(ij)} = \hat{a}_i e^{i\chi\hat{n}_j}, \quad \hat{U}_{\mathrm{cK}}^{(ij)\dagger} \hat{a}_j \hat{U}_{\mathrm{cK}}^{(ij)} = \hat{a}_j e^{i\chi\hat{n}_i}. \tag{63}$$

In view of (63) and (62), we obtain that

$$\begin{pmatrix} \hat{a}_1''' \\ \hat{a}_2''' \\ \hat{a}_3''' \\ \hat{a}_4''' \end{pmatrix} = \hat{U}_3''^\dagger \begin{pmatrix} \hat{a}_1'' \\ \hat{a}_2'' \\ \hat{a}_3'' \\ \hat{a}_4'' \end{pmatrix} \hat{U}_3'' = \begin{pmatrix} \hat{a}_1' e^{i\chi\hat{n}_2''} \\ \hat{a}_2'' e^{i\chi\hat{n}_1'} \\ \hat{a}_3'' e^{i\chi\hat{n}_4'} \\ \hat{a}_4' e^{i\chi\hat{n}_3''} \end{pmatrix}. \tag{64}$$

Based on Eqs. (62) and (64), the Stokes operators after the nonlinear stage have the following form in terms of operators $\hat{a}_i'$,

$$\hat{\mathcal{J}}_{z,0}^{(14)'''} = \frac{1}{2}(\hat{n}_1' \mp \hat{n}_4') = \hat{\mathcal{J}}_{z,0}^{(14)'}, \quad \hat{\mathcal{J}}_{+}^{(14)'''} = \hat{\mathcal{J}}_{+}^{(14)'} e^{-2i\chi\hat{\mathcal{J}}_x^{(23)'}}. \tag{65}$$

The final operators of output modes 1^f and 4^f are found to be

$$\begin{aligned} \hat{a}_1^{(\mathrm{f})} &= \hat{U}_4'''^\dagger \hat{a}_1''' \hat{U}_4''' = c'\hat{a}_1''' - is'\hat{a}_4''', \\ \hat{a}_4^{(\mathrm{f})} &= \hat{U}_4'''^\dagger \hat{a}_4''' \hat{U}_4''' = -s'\hat{a}_1''' - ic'\hat{a}_4''', \end{aligned} \tag{66}$$

where $c' = \cos\theta'$ and $s' = \sin\theta'$ are the parameters of final BS.

From the above expressions we can unitarily relate the Stokes operators of the output (f) to those of the input (in). The population difference operator of the hot output modes 1 and 4 $(\hat{\mathcal{J}}_z^{(14)})^{(\mathrm{f})}$ is determined by the (χ-dependent) exponential of combined $\hat{\mathcal{J}}_x^{(ij)}$ operators of all four modes:

$$(\hat{\mathcal{J}}_z^{(14)})^{(\mathrm{f})} = \frac{-i}{2}\hat{\mathcal{J}}_{+}^{(14)'} e^{-2i\chi\hat{\mathcal{J}}_x^{(23)'}} + \mathrm{H.c.}, \tag{67a}$$

where

$$\hat{\mathcal{J}}_{+}^{(14)'} = \left[c^2\hat{\mathcal{J}}_{+}^{(14)} - s^2\hat{\mathcal{J}}_{+}^{(23)} + cs(\hat{\mathcal{J}}_{+}^{(24)} - \hat{\mathcal{J}}_{+}^{(13)})\right]^{(in)}, \tag{67b}$$

$$\hat{\mathcal{J}}_{x}^{(23)'} = \left[-s^2\hat{\mathcal{J}}_{x}^{(14)} + c^2\hat{\mathcal{J}}_{x}^{(23)} + sc(\hat{\mathcal{J}}_{x}^{(24)} - \hat{\mathcal{J}}_{x}^{(13)})\right]^{(in)}. \tag{67c}$$

The Stokes operators at the output (f) are thus found to be related to their input (in) counterparts in an exactly solvable, intricate nonlinear fashion. For $\chi = 0$, this is a BS-induced LIGO given by Eq. (67b). In contrast, $\chi \neq 0$, the population difference operator $(\hat{\mathcal{J}}_z^{(14)})^{(f)}$ becomes a quantum nonlinear NGO: Eq. (67a) then nonlinearly (exponentially) depends on the intermode correlations as seen from Eq. (67c).

B. Stokes parameters and Poincaré sphere

Consider now the Stokes parameters of modes 1 and 4. The initial wave function is $|\psi\rangle = |\alpha_1\rangle\,|0\rangle\,|0\rangle\,|\alpha_4 e^{i\phi}\rangle$, where $\alpha_1, \alpha_4 > 0$. The Stokes parameters of modes 1 and 4 corresponding to $|\psi\rangle$ are

$$S_x = \alpha_1\alpha_4\cos\phi, \quad S_y = \alpha_1\alpha_4\sin\phi, \quad S_z = \frac{1}{2}(\alpha_1^2 - \alpha_4^2), \quad S_0 = \frac{1}{2}(\alpha_1^2 + \alpha_4^2). \tag{68}$$

Using Eq. (61), we obtain that the beam splitters coupling mode 1 to mode 2 and mode 4 to mode 3 decrease the Stokes parameters of modes 1 and 4 by the factor c^2,

$$\begin{aligned} S'_x &= c^2 S_x, && (69)\\ S'_y &= c^2 S_y, && (70)\\ S'_z &= c^2 S_z, && (71)\\ S'_0 &= c^2 S_0. && (72) \end{aligned}$$

At the same time, the new state of modes 2 and 3 corresponds to a smaller copy of the original Poincaré sphere, which is also rotated by π around the z axis, with the Stokes parameters $-s^2 S_x,\ -s^2 S_y,\ s^2 S_z,\ s^2 S_0$.

It is convenient to average the Stokes operators over the function $|\psi'\rangle = U_1 |\psi\rangle = |\alpha'_1\rangle |\alpha'_2\rangle |\alpha'_3\rangle |\alpha'_4\rangle$.

The BS transformation of the coherent state amplitudes is given by the same matrix B as for the operators a_i, see (52) and (53). Therefore, we obtain

$$\begin{pmatrix} \alpha'_1 \\ \alpha'_2 \\ \alpha'_3 \\ \alpha'_4 \end{pmatrix} = \begin{pmatrix} c\alpha_1 \\ -s\alpha_1 \\ s\alpha_4 e^{i\phi} \\ c\alpha_4 e^{i\phi} \end{pmatrix}, \qquad (73)$$

yielding the Stokes parameters

$$S'''_{z,0} = \langle \psi' | \hat{\mathcal{J}}^{(14)\prime\prime\prime}_{z,0} | \psi' \rangle = \frac{1}{2}(|\alpha'_1|^2 \mp |\alpha'_4|^2) = c^2 S_{z,0}. \qquad (74)$$

To obtain S'''_x and S'''_y, we note that the equality

$$e^{-2i\chi \hat{\mathcal{J}}^{(23)\prime}_x} |\alpha'_2\rangle |\alpha'_3\rangle = |\tilde{\alpha}_2\rangle |\tilde{\alpha}_3\rangle, \qquad (75)$$

is equivalent to a BS transformation,

$$\begin{pmatrix} \tilde{\alpha}_2 \\ \tilde{\alpha}_3 \end{pmatrix} = \begin{pmatrix} c_\chi \alpha'_2 - i s_\chi \alpha'_3 \\ c_\chi \alpha'_3 - i s_\chi \alpha'_2 \end{pmatrix}, \qquad (76)$$

where $c_\chi = \cos\chi$ and $s_\chi = \sin\chi$. In view of (75),

$$S'''_+ = \langle \psi' | \hat{\mathcal{J}}^{(14)\prime\prime\prime}_+ | \psi' \rangle = \alpha'^*_1 \alpha_4 \langle \alpha'_2 | \tilde{\alpha}_2 \rangle \langle \alpha'_3 | \tilde{\alpha}_3 \rangle. \qquad (77)$$

To further transform this expression, we use the equality

$$\langle \alpha' | \alpha \rangle = e^{\alpha'^* \alpha - (|\alpha|^2 + |\alpha'|^2)/2} \qquad (78)$$

and note that $|\tilde{\alpha}_2|^2 + |\tilde{\alpha}_3|^2 = |\alpha'_2|^2 + |\alpha'_3|^2$, since the transformation (76) is unitary. Then, on inserting (73), we obtain finally

$$\begin{aligned} S'''_x &= \mathrm{Re} S'''_+ = c^2 \alpha_1 \alpha_4 e^{-d} \cos(b\cos\phi + \phi), \\ S'''_y &= \mathrm{Im} S'''_+ = c^2 \alpha_1 \alpha_4 e^{-d} \sin(b\cos\phi + \phi), \end{aligned} \qquad (79)$$

where

$$b = 2s^2 \alpha_1 \alpha_4 \sin\chi, \quad d = s^2(1 - \cos\chi)(\alpha_1^2 + \alpha_4^2). \qquad (80)$$

The Stokes parameters (74) and (79) can be recast as functions of the initial Stokes parameters (68),

$$\begin{aligned} S'''_x &= c^2 \exp\left(-4s^2 S_0 \sin^2 \frac{\chi}{2}\right) \left[S_x \cos\left(2s^2 \sin\chi S_x\right) + S_y \sin\left(2s^2 \sin\chi S_x\right)\right], && (81)\\ S'''_y &= c^2 \exp\left(-4s^2 S_0 \sin^2 \frac{\chi}{2}\right) \left[-S_x \sin\left(2s^2 \sin\chi S_x\right) + S_y \cos\left(2s^2 \sin\chi S_x\right)\right], && (82)\\ S'''_z &= c^2 S_z, && (83)\\ S'''_0 &= c^2 S_0. && (84) \end{aligned}$$

This shows that the cross-Kerr induced transformation does not change the Stokes parameters S_z and S_0 but moves the points closer to the z-axis on the Poincare sphere and rotates them by an angle proportional to S_x (see Fig. 2(a)).

Averaging the Stokes parameters (74) and (79) over the random ϕ for the case $\alpha_1 = \alpha_4$ yields

$$\bar{S}_x''' = \bar{S}_z''' = 0, \quad \bar{S}_y''' = c^2 \alpha_1^2 e^{-d} J_1(b), \quad \bar{S}_0''' = c^2 \alpha_1^2, \tag{85}$$

where $J_1()$ is a Bessel function of the first kind. At the steering stage, the point on the Poincaré sphere corresponding to (85) rotates, and we arrive at the expressions in the main text.
